# Successful Rituximab Treatment of GPIHBP1 Autoantibody-Associated Hypertriglyceridemia

**DOI:** 10.1016/j.jaccas.2025.104647

**Published:** 2025-08-13

**Authors:** Tasmeen Hussain, Audra Horomanski, Sneha Jain, Cindy Lamendola, Fahim Abbasi, Masami Murakami, Joshua W. Knowles

**Affiliations:** a Division of Cardiovascular Medicine, Stanford University, Stanford, California, USA; b Division of Immunology and Rheumatology, Stanford University, Stanford, California, USA; c Department of Clinical Laboratory Medicine, Gunma University Graduate School of Medicine, Maebashi, Japan; d Division of Cardiovascular Medicine, Cardiovascular Institute and Diabetes Research Center, Stanford University, Stanford, California, USA

**Keywords:** blood tests, diet, genetics, lipid metabolism disorders, triglycerides

## Abstract

**Background:**

A 50-year-old woman had triglyceride values up to 1640 mg/dL on routine laboratory tests. Despite the initiation of fenofibrate, icosapent ethyl, rosuvastatin, and a low-fat diet, her triglyceride values ranged from 1200 to more than 8200 mg/dL, and she had recurrent pancreatitis.

**Case Summary:**

Testing was negative for mutations in chylomicronemia genes such as *LPL, APOC2, APOA5, LMF1,* and *GPIHBP1.* Additional testing revealed elevated autoantibodies to GPIHBP1 up to 2,336 U/mL (normal <58 U/mL) and decreased GPIHBP1 to 2.5 pg/mL (normal range 570-1,625 pg/mL), confirming GPIHBP1 autoantibody syndrome (GPIHBP1-AAS). The patient received rituximab 1000 mg infusion, with 2 doses given 3 weeks apart. Triglycerides decreased from 1,746 to 81 mg/dL within 4 months and remained normal 12 months later without repeat dosing.

**Discussion:**

GPIHBP1-AAS was only recently described and is associated with severe hypertriglyceridemia and recurrent pancreatitis. In our case, treatment with rituximab was very effective.

**Take-Home Message:**

GPIHBP1-AAS–associated hypertriglyceridemia should be recognized and can be successfully treated with rituximab.

## Visual Summary

**Figure:**
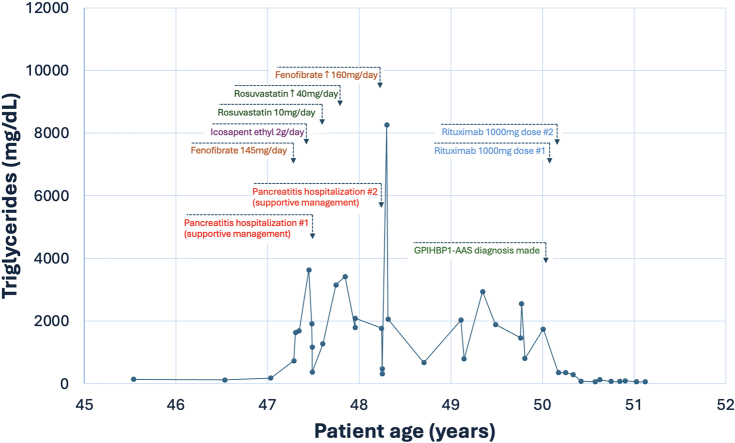

**Visual Summary:**
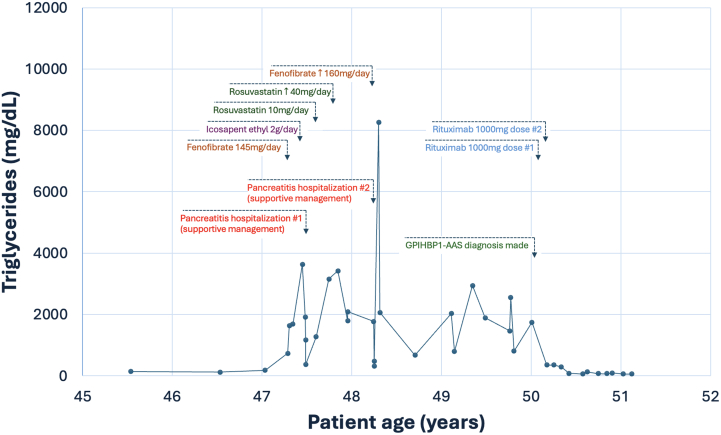
Triglyceride Levels Over Time in a Patient With GPIHBP1 Autoantibody Syndrome Before age 47 years, the patient had relatively normal triglycerides. Between ages 47 and 50 years, her triglycerides rose to more than 8,200 mg/dL while on multiple medicines, and she was hospitalized twice for pancreatitis. Around age 50 years, GPIHBP1 autoantibody syndrome was diagnosed, and she was started on rituximab therapy. Subsequently, her triglycerides returned to normal levels.

## History of Presentation

A 50-year-old woman was noted to have triglycerides up to 1,640 mg/dL on a routine lipid panel. On examination, she had a normal body mass index of 21 and was normotensive; no xanthomas were noted on skin inspection. She was started on fenofibrate 145 mg daily and icosapent ethyl 2 mg twice a day. Despite these treatments, several months later she experienced an episode of severe pancreatitis with triglycerides up to 3,633 mg/dL requiring hospitalization. After discharge, her medical management was intensified to fenofibrate 145 mg daily, icosapent ethyl 2 g twice a day, and rosuvastatin 40 mg daily with a strict low-fat diet. Despite this regimen, over several years her triglycerides remained above 1,200 mg/dL (peak 8,260 mg/dL), and she was hospitalized again for pancreatitis.

**Take-Home Messages**

• Autoantibody testing for GPIHBP1-AAS should be highly considered when work-up for genetic causes of severe hypertriglyceridemia is unrevealing.
• GPIHBP1-AAS should be rapidly diagnosed as it can be successfully treated with rituximab.

## Past Medical History

The patient had a history of moyamoya disease and left internal carotid artery stenosis treated successfully with bypass. She had recently experienced menopause. Notably, in lipid panels more than 1 year before presentation, the patient's triglycerides had been relatively normal (range 76-178 mg/dL [normal 0-149 mg/dL]), and low-density lipoprotein cholesterol ranged from 102 to 155 mg/dL off of lipid-lowering therapies.

Regarding family history, the patient was of Han Chinese ancestry. The patient's maternal grandmother had a history of significant hypertriglyceridemia but did not have a clear history of pancreatitis. No other family members were known to have either hypertriglyceridemia or pancreatitis. There was no family history of heart attack. The patient's paternal grandfather had a stroke after age 70 years. Regarding autoimmune disease, the patient noted that both her mother and a maternal uncle had type 2 diabetes, which “turned into” type 1 diabetes later in life (after age 70 years), and she had a paternal cousin with systemic lupus erythematosus.

## Differential Diagnosis

No secondary cause of hypertriglyceridemia was identified; hemoglobin A_1c_ was 5.7%. Testing was negative for mutations in genes associated with chylomicronemia such as *LPL, APOC2, APOA5, LMF1,* and *GPIHBP1.*

## Investigations

Additional testing (courtesy of Dr M. Murakami, Gunma University) revealed highly elevated autoantibodies to GPIHBP1 at 2,336 U/mL (normal <58 units/mL). She also had markedly depressed levels of GPIHBP1 at 2.5 pg/mL (normal range 570-1,625 pg/mL) and lipoprotein lipase at 6 ng/mL (normal 25-105 ng/mL). The patient was referred to rheumatology for GPIHBP1 autoantibody syndrome (GPIHBP1-AAS).^1^ During this visit, she denied any other symptoms suggestive of overlapping rheumatologic disorders and notable stability in her moyamoya disease for the past 10 years. Laboratory evaluation yielded negative antinuclear antibody and antineutrophil cytoplasmic antibody, normal C3 and C4 levels, erythrocyte sedimentation rate 59 mm/h, C-reactive protein 0.5 mg/dL, and unremarkable urinalysis.

## Management

The patient was prescribed rituximab 1000 mg infusion, with 2 doses given 3 weeks apart. Triglycerides dramatically decreased from 1746 to 345 mg/dL 3 weeks after her initial infusion and to 81 mg/dL within 4 months.

## Outcome and Follow-Up

The patient's triglyceride levels remained normal 12 months from her infusions without a need for repeat dosing. She has had no further episodes of pancreatitis.

## Discussion

GPIHBP1-AAS was first described in 2017.^1^ In 2 patients with hypertriglyceridemia, enzyme-linked immunosorbent assay could not identify recombinant GPIHBP1 added to their serum, suggesting immunoassay interference and an antibody present to GPIHBP1. GPIHBP1 is a protein that stabilizes lipoprotein lipase and shuttles it to its site of action on the capillary lumen. Thus, GPIHBP1 dysfunction and downregulation result in hypertriglyceridemia.^2^

GPIHBP1-AAS coincides with other autoimmune conditions. In a series of 22 patients with GPIHBP1-AAS, 14 had diagnosed or suspected overlapping autoimmune disease (6 with systemic lupus erythematosus; 4 with Hashimoto disease; and several with an assortment of other conditions including rheumatoid arthritis, Sjögren disease, and antiphospholipid syndrome).^2^ The authors noted that IgA class and IgG4 subclass GPIHBP1 autoantibodies were predominant. One interesting case study also noted a case of acquired GPIHBP1-AAS and hypertriglyceridemia in a patient who was receiving interferon beta-1a for multiple sclerosis; once the interferon therapy was stopped, the triglycerides normalized, suggesting to the authors that the interferon “fueled” a proinflammatory milieu.^3^

An interesting question remains regarding the relationship between GPIHBP1-AAS and autoimmune disease with identifiable genetic underpinnings. In a 2024 study by Strøm et al, 132 patients with severe hypertriglyceridemia defined as levels >20 mmol/L (1,770 mg/dL) without mutations in *LPL, APOC2, APOA5, LMF1,* and *GPIHBP1* underwent serum testing for GPIHBP1 autoantibodies.^4^ Of those patients, 33% (44 of 132 patients) noted a personal history of diabetes, thyroiditis, a connective tissue disease, or, more broadly, a rheumatic or autoimmune disease. Though 14 of 132 patients had elevated levels of GPIHBP1 autoantibodies above mean + 2.6 SD of normotriglyceridemic controls, 1 patient had true GPIHBP1-AAS with an autoantibody level 26-fold higher than the mean, markedly reduced lipoprotein lipase (11 ng/mL), and a triglyceride level of 54.3 mmol/L (4,805 mg/dL). This woman had chronic mucocutaneous candidiasis owing to heterozygosity for a pathogenic gain-of-function variant in the *STAT1* gene and endorsed diagnoses of Hashimoto disease, seronegative rheumatoid arthritis, and hemolytic anemia. A larger exploration of this relationship between GPIHBP1-AAS and autoimmune disease could be a focus of future investigation.

Regarding therapy, there is evidence that GPIHBP1-AAS responds to immunosuppressive agents. With the direct role of GPIHBP1 autoantibodies in blocking the shuttle of lipoprotein lipase, there is interest in B-cell depletion therapies (BCDTs) to reduce circulating autoantibodies. BCDTs such as those targeting CD20, CD19, and BAFF are widely used in the treatment of autoimmune diseases, including systemic lupus erythematosus, rheumatoid arthritis, and multiple sclerosis. Notably, BCDTs are thought to work beyond just reducing circulating autoantibodies, as evidenced by patients with clinical improvement without a corresponding decrease in autoantibodies. Proposed autoantibody-independent effects of BCDTs include decreased production of cytokines by B cells and less maintenance of tertiary lymphoid tissues.^5^ In patients with GPIHBP1-AAS, the aforementioned study of 22 patients found that the 5 patients treated with rituximab (an anti-CD20 monoclonal antibody) had normalization of plasma triglycerides.^2^ It is unclear which of the mechanisms of BCDTs were most at play, as post-therapy autoantibody levels were not measured or reported in all cases. The duration of response and need for long-term therapy is also unknown as most cases in the literature do not report outcomes or need for repeat BCDT beyond 1 year. Additional case reports of patients with GPIHBP1-AAS show variable adherence and efficacy to therapies such as steroids and mycophenolate mofetil,^2^^,^^6^^,^^7^ though additional, larger studies and reports of long-term outcomes would be helpful to establish an optimal treatment regimen.

Finally, identification of GPIHBP1-AAS is of substantial prognostic value and indicates the importance of effective treatment. In a study of 116 patients with severe hypertriglyceridemia and hypertriglyceridemia-induced pancreatitis, 15% had GPIHBP1 autoantibodies, and recurrence of pancreatitis over 2 years was much higher in the autoantibody-positive group (35%, 6 of 17) than in the negative group (4%, 4 of 99).^8^ Thus, further exploration of GPIHBP1-AAS and treatments is of substantial clinical importance.

## Conclusions

GPIHBP1-AAS has been described only recently and is associated with severe hypertriglyceridemia and recurrent pancreatitis in the literature. In our case, treatment with rituximab was very effective, highlighting the importance of high suspicion and rapid diagnosis of this entity. Autoantibody testing should be considered when work-up for genetic causes of severe hypertriglyceridemia is unrevealing.

## Funding Support and Author Disclosures

The authors have reported that they have no relationships relevant to the contents of this paper to disclose.
